# Spatiotemporal coordination of trophoblast and allantoic Rbpj signaling directs normal placental morphogenesis

**DOI:** 10.1038/s41419-019-1683-1

**Published:** 2019-06-05

**Authors:** Jinhua Lu, Weiwei Wu, Qiliang Xin, Chan Zhou, Jianqi Wang, Zhangli Ni, Dong Liu, Yingchun Xu, Yongqin Yu, Ningjie Yang, Yang Sun, Bo He, Shuangbo Kong, Shumin Wang, Chao Wang, Haibin Wang

**Affiliations:** 1 Reproductive Medical Center, The First Affiliated Hospital of Xiamen University, 361003 Xiamen, Fujian People’s Republic of China; 2 Fujian Provincial Key Laboratory of Reproductive Health Research, School of Medicine, Xiamen University, 361102 Xiamen, Fujian People’s Republic of China; 3 Anti-aging & Regenerative Medicine Research Institution, College of Life Sciences, Shandong University of Technology, 255049 Zibo, Shandong People’s Republic of China; 4 State Key Laboratory of Agrobiotechnology, College of Biological Sciences, China Agricultural University, 100193 Beijing, People’s Republic of China; 5 National Engineering Laboratory for Animal Breeding, Key Laboratory of Animal Genetics, Breeding and Reproduction of the Ministry of Agriculture, College of Animal Science and Technology, China Agricultural University, 100193 Beijing, People’s Republic of China

**Keywords:** Morphogenesis, Reproductive biology

## Abstract

The placenta, responsible for the nutrient and gas exchange between the mother and fetus, is pivotal for successful pregnancy. It has been shown that Rbpj, the core transcriptional mediator of Notch signaling pathway, is required for normal placentation in mice. However, it remains largely unclear how Rbpj signaling in different placental compartments coordinates with other important regulators to ensure normal placental morphogenesis. In this study, we found that systemic deletion of *Rbpj* led to abnormal chorioallantoic morphogenesis and defective trophoblast differentiation in the ectoplacental cone (EPC). Employing mouse models with selective deletion of *Rbpj* in the allantois versus trophoblast, combining tetraploid aggregation assay, we demonstrated that allantois-expressed Rbpj is essential for chorioallantoic attachment and subsequent invagination of allantoic blood vessels into the chorionic ectoderm. Further studies uncovered that allantoic Rbpj regulates chorioallantoic fusion and morphogenesis via targeting *Vcam1* in a Notch-dependent manner. Meanwhile, we also revealed that trophoblast-expressed Rbpj in EPC facilitates Mash2’s transcriptional activity, promoting the specification of *Tpbpα*-positive trophoblasts, which differentiate into trophoblast subtypes responsible for interstitial and endovascular invasion at the later stage of placental development. Collectively, our study further shed light on the molecular network governing placental development and functions, highlighting the necessity of a spatiotemporal coordination of Rbpj signaling for normal placental morphogenesis.

## Introduction

The placenta is vital to the survival and growth of the fetus before birth, since it provides the fetus with nutrients and gases during pregnancy. Any disturbance to placental development would lead to pregnancy-related complications, such as preeclampsia and intrauterine growth restriction^1–3^. In mice, the mature placenta is composed of three distinct cellular layers: the outmost maternal decidua, derived from uterine stromal cells across which the maternal blood comes into the placenta through spiral arteries; the intermediate junction zone, through which the maternal vessels get in and out of the underlying labyrinth layer; the innermost labyrinth layer, highly branching to form large surface areas responsible for nutrient and gas exchanges between the mother and fetus^4^. During placental morphogenesis in mice, the allantois derived from the extraembryonic mesoderm and the chorion developed from the extraembryonic ectoderm undergo chorioallantoic fusion at E8.5 to initiate chorioallantoic branching morphogenesis^5,6^, during which fetal vessels invaginate into the chorionic plate and branch with the underlying trophoblast cells to form placental labyrinth^7^. Simultaneously, maternal spiral arteries (SPA) delivering blood to the placenta undergo intensive remodeling, during which the endothelial cells, even regions of the smooth muscle wall, are replaced by invasive trophoblast cells. Moreover, the glycogen trophoblast cells invade interstitially into maternal decidua and form wrapping layers around spiral arteries^8^, contributing to spiral artery dilation^9^. These dynamic cellular events coordinately decrease vascular resistance of spiral arteries, thus increasing placental perfusion by maternal blood to meet the increasing needs of the fetus^10,11^. However, it remains largely unknown how these developmental processes are tightly coordinated to guarantee normal development and functions of the placenta.

Previous studies have demonstrated that a wide range of molecules in development-related signaling pathways is involved in regulation of placental development, including the molecules in Notch signaling^12–14^. Notch signaling is initiated by the release of the Notch intracellular domain (NICD) when Notch receptors are bound by its specific ligands. When translocated to the nucleus, NICD interacts directly with Rbpj, together with Mastermind-like protein (MAML) to exert transcriptional activity^15^. Rbpj, the nuclear transducer of Notch signaling, is an important transcription factor highly conserved from *Drosophila* to human^16^. It has been reported that systemic deletion of Rbpj led to defective chorioallantoic fusion during early placentation^17^. However, it remains elusive how Rbpj-mediated signaling directs normal chorioallantoic fusion. Moreover, it is still largely unknown about the precise function of Rbpj signaling during trophoblast specification, due to the early death of *Rbpj*-null mutants.

Employing multiple mouse models, we provided herein molecular and genetic evidence that allantoic Rbpj regulates chorioallantoic fusion and branching morphogenesis via targeting *Vcam1* in a Notch-dependent manner. Meanwhile, trophoblast Rbpj facilitates Mash2’s transcriptional activity to promote specification of *Tpbpα*-positive trophoblasts, which differentiate into trophoblast cells responsible for interstitial and endovascular invasion at the later stage of placental development.

## Results

### *Rbpj* deficiency derails normal placental development

**Fig. 1:**
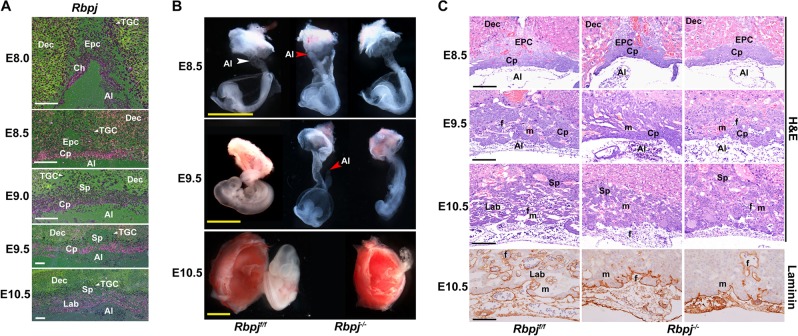
*Rbpj* deficiency derails normal placental development. **a** In situ hybridization analysis of *Rbpj* mRNA expression during early placental development from E8.0 to E10.5. White arrowheads indicate TGCs. Images are representative of two independent experiments. **b** Morphological appearance of the conceptus dissected and removed from the parietal yolk sac at E8.5, and partially dissected feto-placental units at E9.5 and E10.5. The white arrowhead indicates the fused allantois, while the red arrowheads indicate the ends of the allantois failing to fuse with the chorion and appearing as a bud in *Rbpj*^−/−^ mutants. **c** Histological sections of *Rbpj*^f/f^ and *Rbpj*^−/−^ chorionic plates and allantois with hematoxylin–eosin (HE) and Laminin staining from E8.5 to E10.5. The fetal vessels were indicated by Laminin staining. Images in **b** and **c** are representative of at least three independent experiments. Al allantois, Ch chorion, Cp chorion plate, Epc ectoplacental cone, Dec decidual basalis, TGC trophoblast giant cell, Lab labyrinth, Sp spongiotrophoblast layer, f fetal blood vessel, m maternal blood sinus. Yellow scale bars: 1 mm; white and black scale bars: 100 μm

Our previous study has demonstrated that uterine Rbpj is essential for normal embryo development via instructing the initial embryonic–uterine orientation and ensuring normal decidual patterning in a stage-specific manner^18^. To further explore the pathophysiological significance of Rbpj signaling during early placental development, we first performed in situ hybridization to examine the spatiotemporal expression profile of *Rbpj* in extraembryonic tissues. At E8.0, *Rbpj* mRNA expression was detected in the floating allantois and chorion, and also in the ectoplacental cone (EPC), as well as trophoblast giant cells (TGC) (Fig. 1a). With the initiation and progression of chorioallantoic fusion and branching morphogenesis, *Rbpj* expression sustained in chorioallantoic cells at E8.5 followed by expression in placental labyrinth at E9.0–10.5, as well as in EPC and TGCs (Fig. 1a). By quantitative RT-PCR analysis, we confirmed *Rbpj* expressing in isolated allantois and chorion plus EPC (Fig. S1a). The spatiotemporal *Rbpj* expression indicates that Rbpj might play critical roles during chorioallantoic morphogenesis, as well as trophoblast differentiation at the fetal–maternal interface.

To determine the physiological significance of Rbpj during placental development, we employed mouse models with systemic *Rbpj* deletion (*Rbpj*^−/−^) achieved by crossing *Rbpj*^*f*/*f*^/*Zp3*^*Cre*/+^ female mice with *Rbpj*^*f*/*f*^/*Prm*^*Cre*/+^ male mice. At E9.5, *Rbpj*^−/−^ embryos exhibited growth-arrested and possessed pale yolk sacs with disorganized blood vessels, as well as few fetal blood cells (Fig. S1b), consistent with previous observations^17^. In *Rbpj*^f/f^ conceptus, the allantois (Al, white arrowhead) attached to the chorion normally at E8.5 followed by formation of the umbilical artery and vein at E10.5. However, *Rbpj*^−/−^ mutants exhibited defective chorioallantoic attachment and fusion with the allantois (red arrowheads) failing to fuse with the chorion and appearing as a bud at E8.5 and even E9.5, leading to fetal death (Fig. 1b). HE and laminin staining further revealed that Rbpj deletion resulted in defective chorioallantoic fusion and shallow invagination of allantoic blood vessels into the chorionic ectoderm, leading to impaired chorioallantoic branching morphogenesis and labyrinth development, with no or less fetal blood vessels appearing in the placental labyrinth at E10.5 (Fig. 1c and S1c, d). These findings indicate that Rbpj deficiency derails normal chorioallantoic fusion and branching morphogenesis during placentation.

### *Rbpj* in allantois is indispensable for normal chorioallantoic fusion

**Fig. 2:**
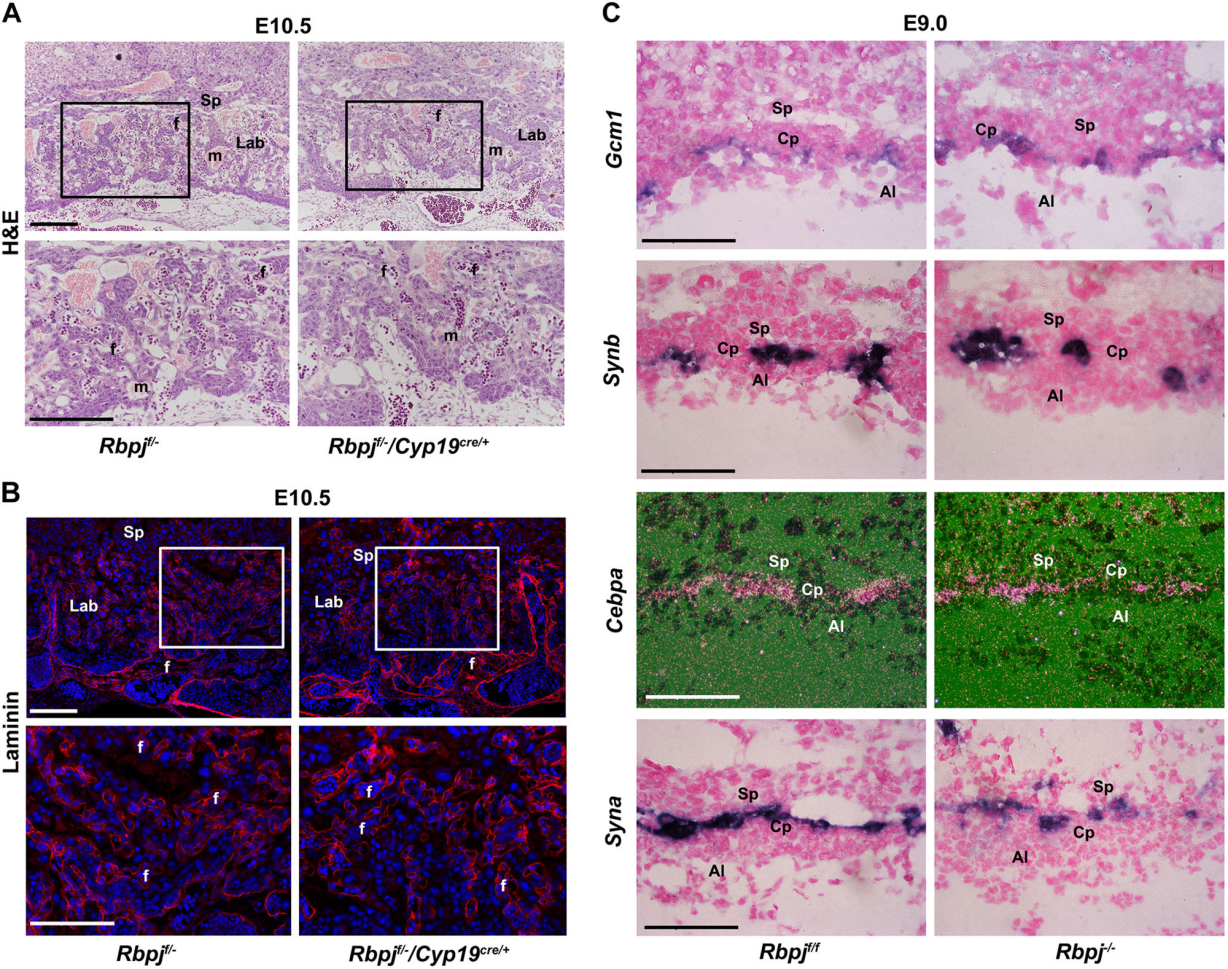
Trophoblast-expressed *Rbpj* is dispensable for chorioallantoic fusion and branching morphogenesis. HE (**a**) and Laminin staining (**b**) of E10.5 placenta with *Rbpj* deleted specifically in placental trophoblast cells by *Cyp19*^*cre*/+^ transgenic mice model. In order to achieve efficient Rbpj deletion in trophoblast cells, we first deleted one allele of Rbpj systemically before deleting the other allele by *Cyp19*^*cre*/+^. Cy3-labeled laminin is in red, DAPI-labeled nuclei in blue. Regions of interest are boxed in black (**a**) or white (**b**), and magnified below. **c** The differentiation of the chorionic trophoblast was revealed by the expressions of *Gcm1*, *Synb*, and *Cebpα* (markers for SynT-II), and *Syna* (marker for SynT-I) in *Rbpj*^f/f^ and *Rbpj*^−/−^ chorion plate (Cp). The expression of these markers was comparable between the control (*Rbpj*^f/f^) and *Rbpj-*null mutants in in situ hybridization assay. Images in **a**–**c** are representative of at least three independent experiments. Al allantois, Cp chorion plate, Lab labyrinth, Sp spongiotrophoblast layer; f fetal blood vessel; m, maternal blood sinus. White and black scale bars: 100 μm

Chorioallantoic attachment and branching morphogenesis require intimate interaction between chorionic trophoblast and allantoic cells^2^. Since Rbpj was expressed in both the allantois and chorion, to ascertain the respective contribution of trophoblast versus allantoic Rbpj to chorioallantoic fusion and branching, we employed the trophoblast-specific *Cyp19*^*cre*/+^ mouse model. In order to achieve efficient Rbpj depletion, one allele of Rbpj was first deleted systemically and then the other was deleted via *Cyp19*^*cre*/+^. As shown in Fig. S2a–c, Rbpj expression was deleted specifically and efficiently in trophoblast cells, not in fetal cells (*Rbpj*^*f*/−^/*Cyp19*^*cre*/+^ placenta). Upon trophoblast-specific Rbpj deletion, chorioallantoic attachment and fetal vascular development occurred normally in *Rbpj*^*f*/−^/*Cyp19*^*cre*/+^placentas, comparable with that of the controls (*Rbpj*^*f*/*f*^ and *Rbpj*^*f*/−^) (Fig. 2a, b). These observations suggest that Rbpj located in the chorionic trophoblast is not required for chorioallantoic fusion and branching morphogenesis.

After chorioallantoic fusion at E8.5, chorionic trophoblast cells coordinate with the allantoic mesoderm and the underlying fetal vessels to initiate primary branching in the chorioallantoic placenta. We then analyzed the expression of marker genes involved in chorionic trophoblast differentiation in *Rbpj*^*f*/*f*^ and *Rbpj*^−/−^ placentas with chorioallantoic fusion. At E9.0, *Gcm1*, which defines branching points and promotes chorionic trophoblast differentiation into syncytiotrophoblast layer II (SynT-II)^19,20^, was expressed normally at branching points in both *Rbpj*^*f*/*f*^ and *Rbpj*^−/−^ placentas. Accordingly, the expressions of *Synb* and *Cebpα*, downstream targets of *Gcm1* and markers for SynT-II, were comparable in both *Rbpj*^*f*/*f*^ and *Rbpj*^−/−^ placentas (Fig. 2c and S2g). Moreover, the expression of *Syna* localized to SynT-I^21^, was not disturbed by Rbpj deletion (Fig. 2c and S2g). These results reinforce the notion that trophoblast-expressed *Rbpj* is dispensable for chorioallantoic trophoblast differentiation and branching morphogenesis.

**Fig. 3:**
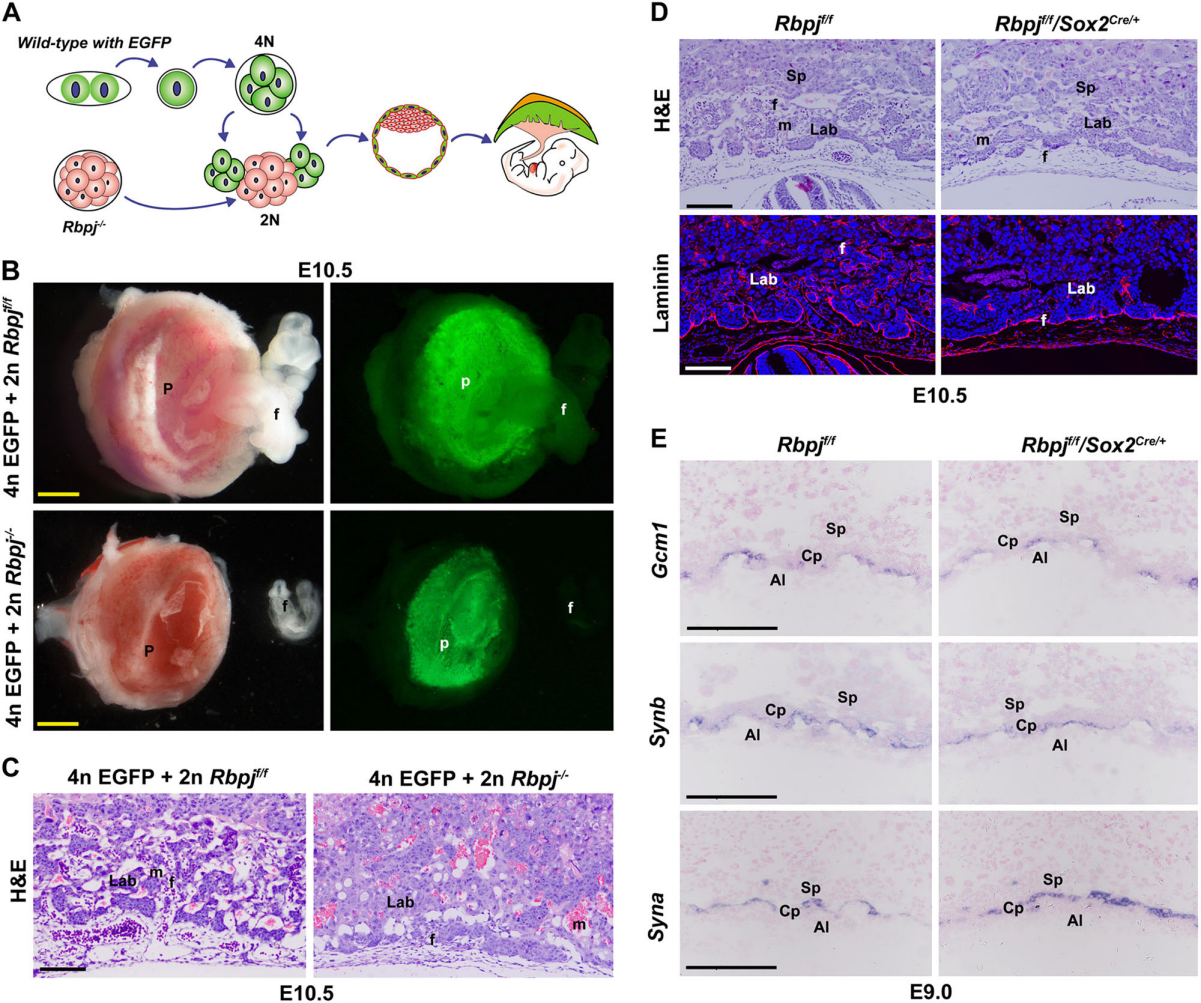
Allantois-expressed *Rbpj* is essential for chorioallantoic fusion and branching morphogenesis. **a** Diagram illustrating the procedure of tetraploid aggregation, in which one *Rbpj*^−/−^ morula aggregated with two wild-type tetraploid EGFP-expressing four-cell embryos. The tetraploid cells contribute exclusively to the trophoblast cells of the placenta, rather than the allantois. **b** E10.5 embryos and placentas generated by tetraploid aggregation assay. **c** HE staining of E10.5 reconstructed placentas from tetraploid aggregation assay. **d** Shallow invagination of allantoic blood vessels into the chorionic ectoderm was revealed by HE and laminin staining in placentas with allantois-specific deletion of *Rbpj* by *Sox2*^*cre*/+^. Cy3-labeled Laminin is in red, DAPI-labeled nuclei in blue. **e** The differentiation of a chorionic trophoblast was revealed by the expressions of *Gcm1* and *Synb* (markers for SynT-II), and *Syna* (marker for SynT-I). By in situ hybridization, the expression of these markers was comparable between the *Rbpj*^f/f^ and *Rbpj*^f/f^/*Sox2*^*cre*/+^ placentas. Images in **b–e** are representative of at least three independent experiments. Al allantois, Cp chorion plate, Lab labyrinth, f fetal blood vessel, m maternal blood sinus, p placenta, Sp spongiotrophoblast layer. Yellow scale bars: 1 mm; white and black scale bars: 100 μm

Based on the aforementioned observations, we surmised that allantois-expressed *Rbpj* could be a vital player directing normal chorioallantoic fusion. To test this hypothesis, we first performed tetraploid aggregation assay, via which the *Rbpj*^−/−^ embryos were aggregated with wild-type tetraploid embryos with EGFP expression (Fig. 3a). Tetraploid cells contribute exclusively to trophoblast cells of the placenta and endoderm of the yolk sac, rather than to allantois. As illustrated in Fig. 3b, c, while the EGFP-expressing cells derived from wild-type tetraploid embryos contributed exclusively to *Rbpj*^*f*/*f*^ placentas, as well as *Rbpj*^−/−^ placentas at E10.5, impaired chorioallantoic fusion and branching were not rescued in the chimera conceptus. These findings clearly demonstrate that allantois-expressed Rbpj is essential for normal chorioallantoic fusion and branching morphogenesis. To verify this finding, we employed *Sox2*^*cre*/+^ mouse model to delete *Rbpj* selectively in the epiblast, and therefore in extraembryonic mesoderm-derived allantois, as well as fetal vessels in the placenta^22^ (Fig. S3a, b). While growth arrest and defective chorioallantoic morphogenesis were observed in *Rbpj*^*f*/*f*^/*Sox2*^*cre*/+^ placenta (Fig. 3d and S3c), the expression of markers involved in chorionic trophoblast differentiation was unaffected (Fig. 3e). These data further reinforce the notion that allantois-expressed Rbpj is essential for normal chorioallantoic fusion and branching morphogenesis.

### Rbpj directs allantoic *Vcam1* expression in a Notch-dependent manner

**Fig. 4:**
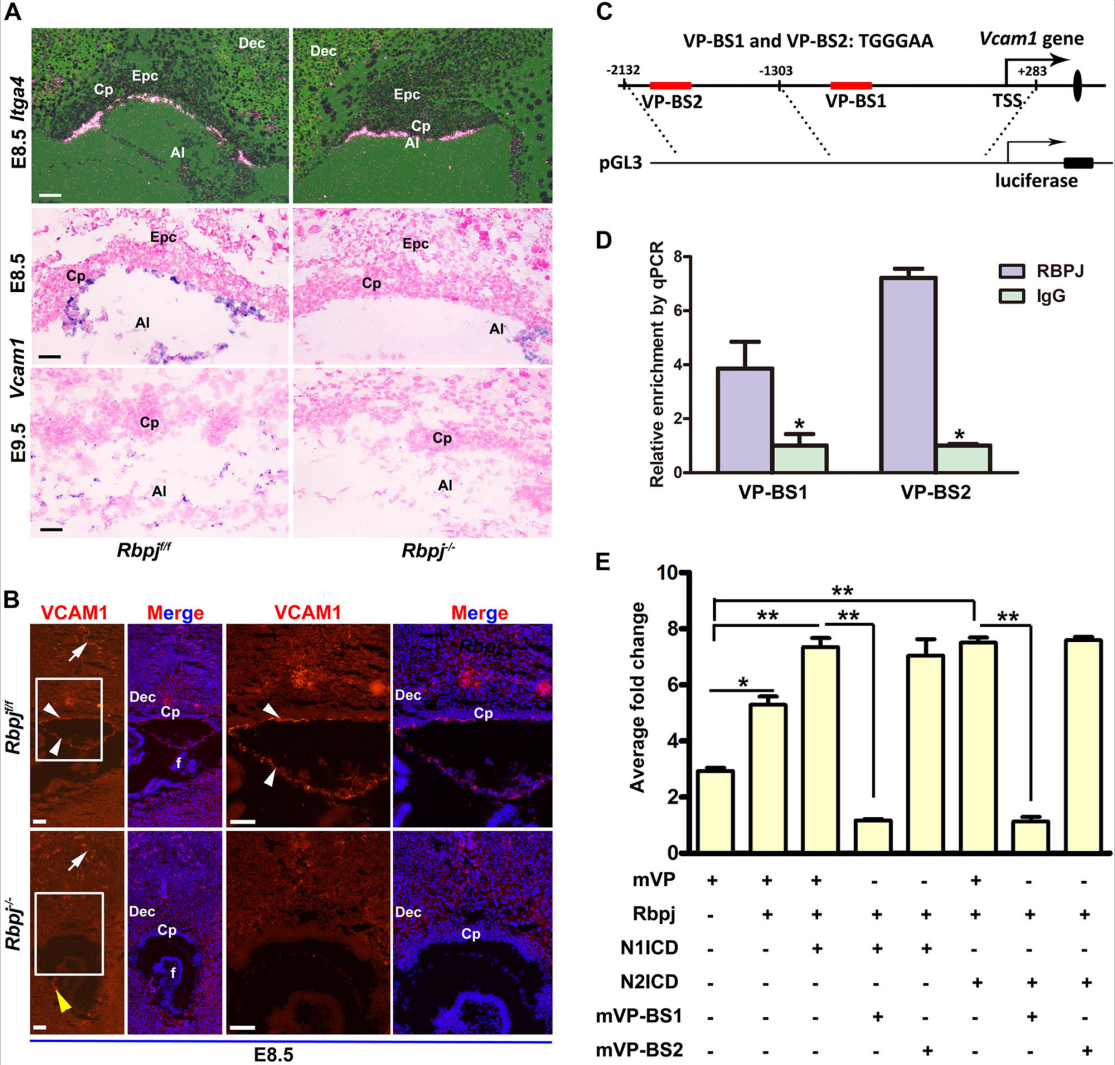
Rbpj directs allantoic *Vcam1* expression in a Notch-dependent manner. **a** The expression of *Itga4* and *Vcam1* was detected by in situ hybridization in the controls (*Rbpj*^f/f^) versus *Rbpj*^−/−^ mutants. **b** VCAM1 expression at protein level was revealed in E8.5 control (*Rbpj*^*f*/*f*^) and *Rbpj*^−/−^ chorion plate. Cy3-labeled VCAM1 is in red, DAPI-labeled nuclei in blue. White arrows indicate VCAM1 expression in maternal decidual vascular endothelial cells. White arrowheads, the VCAM1 expression in allantoic cells. Yellow arrowheads, the VCAM1 expression in the fetus. Regions of interest are boxed in white and magnified on the right. **c** Schematic representation of the promoter region of *Vcam1* and PGL3-Vcam1 constructs. The red rectangle indicated two potential *Rbpj*-binding sites within a region spanning 2 kb upstream of the TSS of *Vcam1*. **d** ChIP assay revealed that Rbpj was enriched at the promoter region of *Vcam1* in E8.5 placentas. *N* = 3. **e** The activity of the *Vcam1* promoter was activated by overexpression of *Rbpj* in combination with NICD (NICD1 or NICD2) overexpression or not. Mutation analysis revealed that only VP-BS1 responded to the activation of *Rbpj*-mediated Notch signaling. *N* = 3. *P < 0.05, **P < 0.01. Images in **a** and **b** are representative of at least three independent experiments. Al allantois, Cp chorion plate, Dec decidual basalis, Epc ectoplacental cone, f fetus. Scale bars: 100 μm

Chorion–allantois fusion is based on cell–cell contact that is mediated by a variety of cell adhesions and signaling molecules. The vascular cell adhesion molecule-1 (*Vcam1*), expressed on the distal two-thirds of allantois, and α4 integrin (*Itga4*) localized to the chorionic basal side, lie at a pivotal step during chorioallantoic attachment and fusion, since deletion of each led to defective chorioallantoic fusion^23,24^. To search for the underlying molecular basis intrinsic to allantoic defects of *Rbpj* mutants, we examined the expression of *Itga4* and *Vcam1* in *Rbpj*^*f*/*f*^ and *Rbpj*^−/−^ placentas. While *Itga4* expression was detectable in both the *Rbpj*^*f*/*f*^ and *Rbpj*^−/−^ basal chorions, the expression of *Vcam1* almost disappeared in *Rbpj*^−/−^ placentas at E8.5 and E9.5 (Fig. 4a, b). These findings suggest a potentially functional association between Rbpj and Vcam1 during chorioallantoic attachment and branching. However, it remained unclear how allantoic Rbpj ensures normal Vcam1 expression during early placentation.

Since *Rbpj* is a transcriptional factor^25^, we speculated that Vcam1 could be a direct target gene of Rbpj. In order to test this hypothesis, we searched the promoter region of *Vcam1* and found two prospective binding sites of Rbpj (Fig. 4c), located at −1006 to −1000 bp (VP-BS1) and −1824 to −1818 bp (VP-BS2) upstream of the transcription start site (TSS), respectively. Chromatin immunoprecipitation (ChIP) assay using antibodies against Rbpj revealed an enriched accumulation of Rbpj at the two binding sites in E8.5 placenta (Fig. 4d). Luciferase assay further revealed that VP-BS1 was responsive to *Rbpj* overexpression, while its mutant form abrogating the conserved Rbpj-binding sequence (mVP-BS1) failed to respond to *Rbpj* signaling (Fig. 4e). Since *Notch1* and *Notch2* were expressed in allantois (Fig. S4) and the phenotype of Notch1-deficient mice was similar to that of Rbpj-deficient mice^26^, we thus surmised that *Notch1* and *Notch2* could be the prospective receptors driving *Rbpj* signaling to regulate *Vcam1* expression during chorioallantoic fusion. Indeed, luciferase activity driven by Vcam1 promoter was increased prominently when N1ICD (Notch1) and N2ICD (Notch2) were cotransfected with *Rbpj*, respectively (Fig. 4e). The results suggest that Rbpj signaling ensures normal chorioallantoic fusion and branching morphogenesis via directly inducing Vcam1 expression in a Notch-dependent manner.

**Fig. 5:**
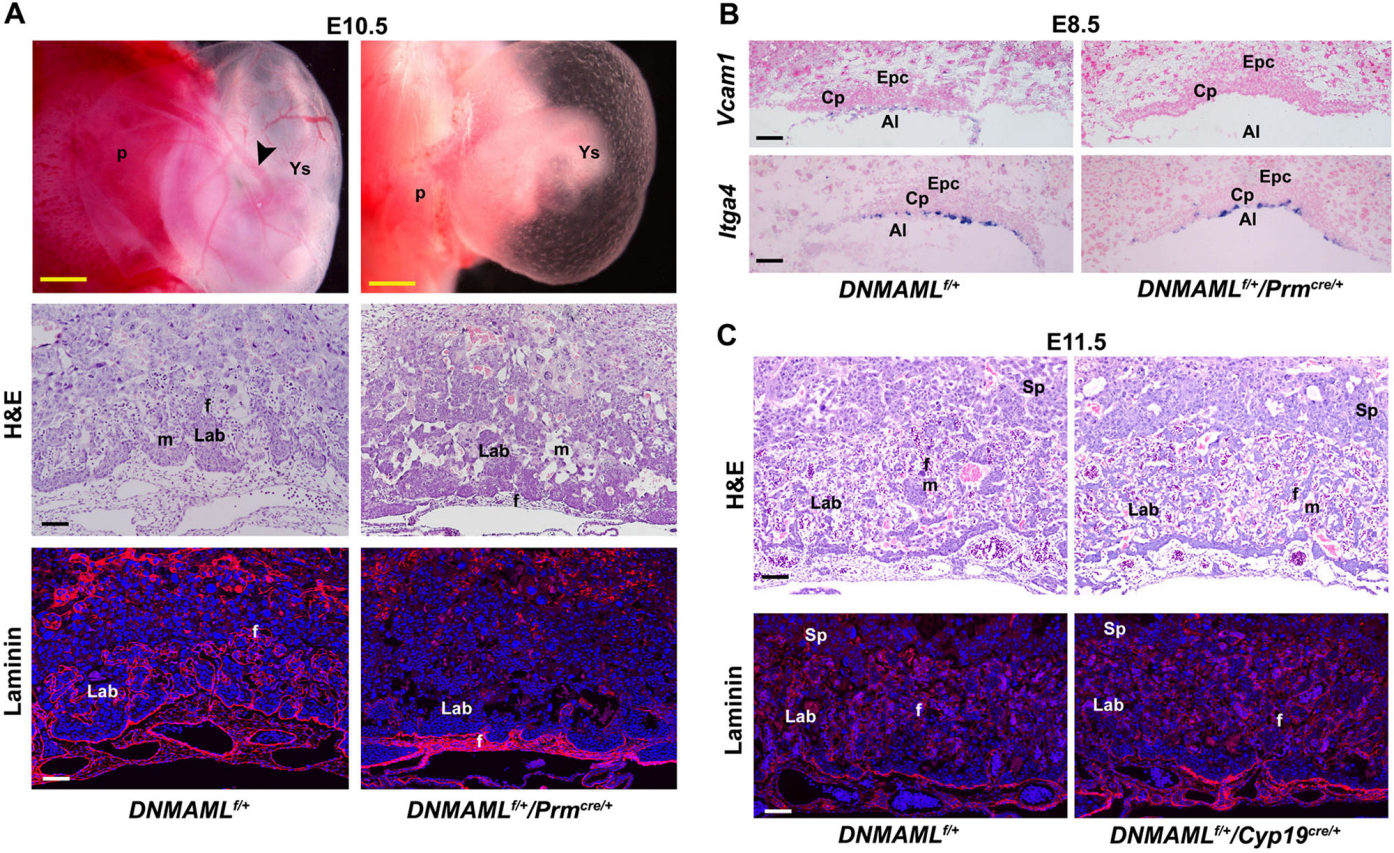
Genetic evidence for *Rbpj*-mediated Notch signaling pathway regulating chorioallantoic morphogenesis. **a** Whole-mount views, as well as HE and Laminin staining of *DNMAML*^*f*/+^ and *DNMAML*^*f*/+^/*Prm*^*cre*/+^ placentas and yolk sacs. Note that the expression of dominant negative MAML (DNMAML), which blocks Notch-Rbpj signaling, disturbs normal chorioallantoic branching morphogenesis. Black arrowheads indicated the large vitelline vessels in the yolk sac. Cy3-labeled Laminin is in red, DAPI-labeled nuclei in blue. **b** In situ hybridization analysis of *Itga4* and *Vcam1* expression. Note the disappeared expression of *Vcam1* in the allantois of *DNMAML*^*f*/+^/*Prm*^*cre*/+^ placentas. **c** HE and laminin staining of *DNMAML*^*f*/+^ and *DNMAML*^*f*/+^/*Cyp19*^*cre*/+^ placentas. Note the well-intermingled maternal sinuses and the invading fetal vessels filled with nucleated erythrocytes in E10.5 *DNMAML*^*f*/+^/*Cyp19*^*cre*/+^ placentas. Cy3-labeled Laminin is in red, DAPI-labeled nuclei in blue. Images in **a**–**c** are representative of at least three independent experiments. Al allantois, Cp chorion plate, Lab labyrinth, Sp spongiotrophoblast, f fetal blood vessel, m maternal blood sinus, p placenta, Ys yolk sac. Yellow scale bars: 1 mm; white and black scale bars: 100 μm

To further confirm that the participation of *Rbpj* signaling in chorioallantoic morphogenesis is driven by canonical Notch activation, we employed *DNMAML*^*f*/+^/*Prm*^*cre*/+^ and *DNMAML*^*f*/+^/*Cyp19*^*cre*/+^ mouse models with haploid overexpression of dominant negative MAML (DNMAML), which blocks canonical Notch signaling^27^. As shown in Fig. 5a, defective chorioallantoic development was observed in *DNMAML*^*f*/+^/*Prm*^*cre*/+^ placentas, similar to that of *Rbpj*^−/−^ placentas. Most importantly, *Vcam1* expression was almost diminished in *DNMAML*^*f*/+^/*Prm*^*cre*/+^ allantois at E8.5, while *Itga4* was expressed normally (Fig. 5b). Moreover, as expected, normal labyrinth formation was observed in *DNMAML*^*f*/+^/*Cyp19*^*cre*/+^ placenta, in which DNMAML functions specifically in trophoblast cells (Fig. 5c), consistent with the recent findings^28^, further demonstrating that Rbpj-mediated canonical Notch signaling in chorionic trophoblast cells is dispensable for chorioallantoic attachment and branching. These data provided a new line of genetic evidence that allantoic *Rbpj* directs *Vcam1* expression in a Notch-dependent manner.

### *Rbpj* deficiency attenuates specification of *Tpbpα*-positive trophoblast in EPC

**Fig. 6:**
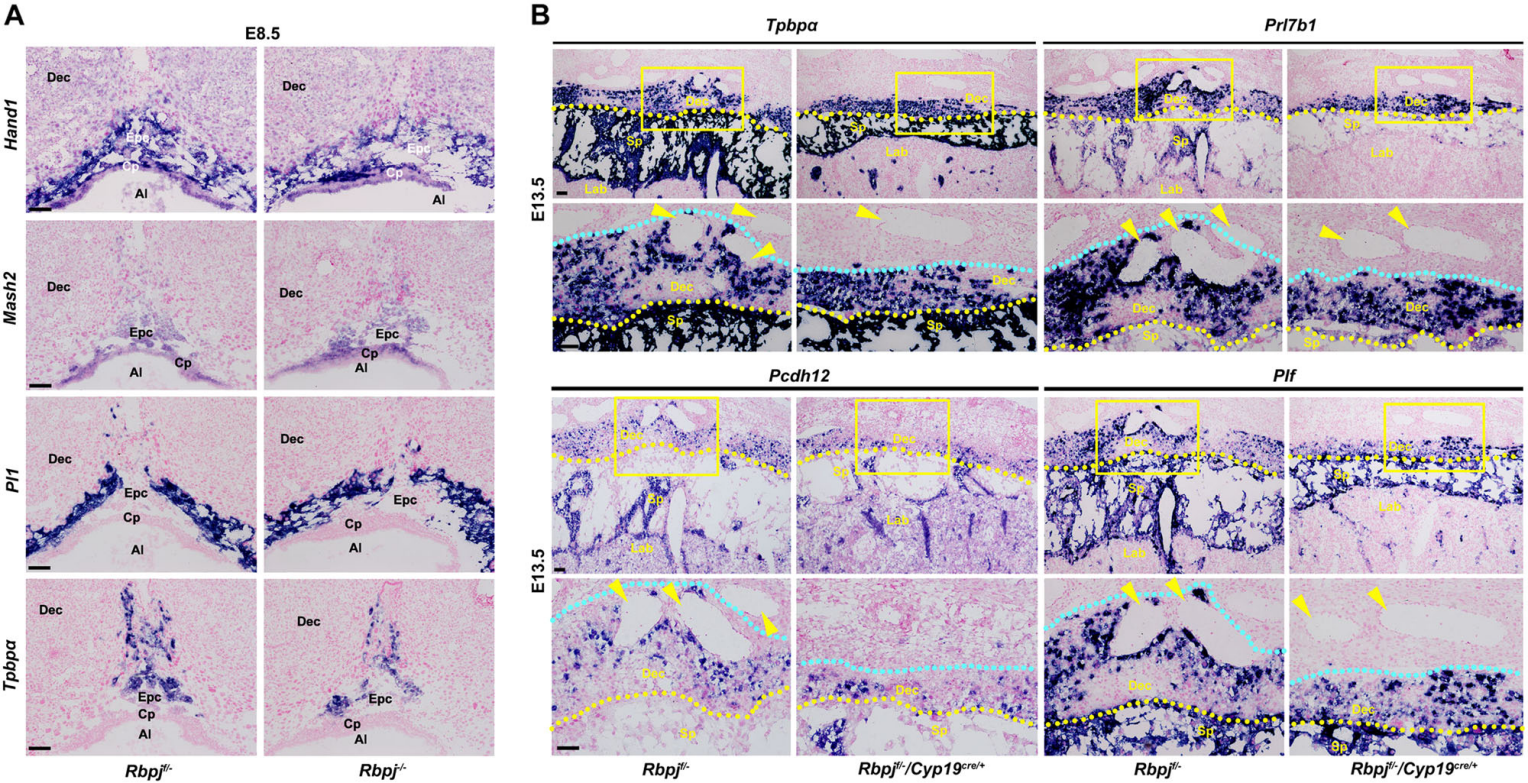
*Rbpj* deficiency attenuates the specification of *Tpbpα*-positive trophoblast. **a** The expression of trophoblast marker genes was detected by in situ hybridization analysis in the control (*Rbpj*^f/−^) versus *Rbpj-*null ectoplacental core at E8.5. The *Hand1* expression was unchanged by *Rbpj* deletion, correlating with normal differentiation of Pl1-expressing giant cells. While the expression of *Mash2* was unaffected by *Rbpj* deficiency, the *Tpbpα*-positive spongiotrophoblast cells were decreased significantly. **b** In situ hybridization analysis revealed that the invasive ability of trophoblast cells differentiated from the *Tpbpα*-positive trophoblast cells in the ectoplacental core was disturbed by *Rbpj* deletion. *Prl7b1* marks both the invasive glycogen trophoblast cells and SPA-TGCs. *Pcdh12* marks glycogen trophoblast cells and *Plf* marks SPA-TGCs. Yellow arrowheads indicate the maternal spiral arteries. Yellow dotted lines indicate the interface between the maternal decidua and the spongiotrophoblast layer, while the light blue dotted lines show the boundaries that the trophoblast invades to the maternal decidua. The distance of trophoblast invasion is between the yellow and light blue lines. Regions of interest are boxed in yellow and magnified below. Images in **a** and **b** are representative of at least three independent experiments. Al allantois, Cp chorion plate, Epc ectoplacental cone, Dec decidual basalis, Lab labyrinth, Sp spongiotrophoblast. Scale bars: 100 μm

Regardless of the dispensability of Rbpj signaling in chronic trophoblast differentiation, we eventually looked into the significance of Rbpj during ectoplacental cone development, since its expression was persistent in EPC and TGCs at E8.0–10.5 (Fig. 1a). We analyzed mRNA expression of marker genes important for EPC development. The expression of heart and neural crest derivatives expressed transcript 1 (*Hand1*), an essential regulator of TGC specification^29^, was comparable between *Rbpj*^f/−^ and *Rbpj*^−/−^ placentas (Fig. 6a). This observation well correlated with the normal differentiation of *Pl1*-expressing TGCs in *Rbpj*^−/−^ placentas (Fig. 6a). By contrast, while the expression of achaete–scute complex homolog-like 2 (Ascl2, also Mash2), known to stimulate specification of the spongiotrophoblast lineage^30^, was not disturbed, the population of trophoblast-specific protein α (*Tpbpα*)-positive cells was decreased significantly by *Rbpj* deletion (Fig. 6a). These findings suggest that Rbpj deletion attenuates specification of *Tpbpα*-positive trophoblast in EPC.

Since *Tpbpα*-positive trophoblast progenitors could differentiate into spongiotrophoblast cells, glycogen trophoblast cells, as well as endovascular TGCs that invade into the maternal decidua and SPA, respectively, beyond mid-gestation^31,32^, we wonder whether attenuated specification of *Tpbpα*-positive trophoblast would hamper the functions of mature placenta, particularly trophoblast invasion into the maternal decidua and SPA. Indeed, the E13.5 fetuses with *Rbpj*^*f*/−^/*Cyp19*^*cre*/+^ placentas were a little pale, compared with those of control placentas (Fig. S5a). Moreover, the weight of the fetuses with *Rbpj*^*f*/−^/*Cyp19*^*cre*/+^ placenta was decreased significantly, while the placental weight was unaffected (Fig. S5b). We next analyzed the differentiation of *Tpbpα*-positive trophoblast in E13.5 placentas. As illustrated in Fig. 6b, *Tpbpα*-positive spongiotrophoblast population was reduced significantly upon *Rbpj* deficiency (*Rbpj*^*f*/−^/*Cyp19*^*cre*/+^). More importantly, the population and invasion of glycogen trophoblasts expressing *Prl7b1* and *Pcdh12* were reduced substantially in *Rbpj*^*f*/−^/*Cyp19*^*cre*/+^placentas (Fig. 6b). Moreover, the differentiation and invasion of *Prl7b1*-positive and *Plf*-positive TGCs were disturbed by Rbpj deletion (Fig. 6b). These results indicate that intact Rbpj signaling is essential for normal specification of *Tpbpα*-positive trophoblast cells.

### Rbpj facilitates Mash2 to promote specification of Tpbpα-positive trophoblast

**Fig. 7:**
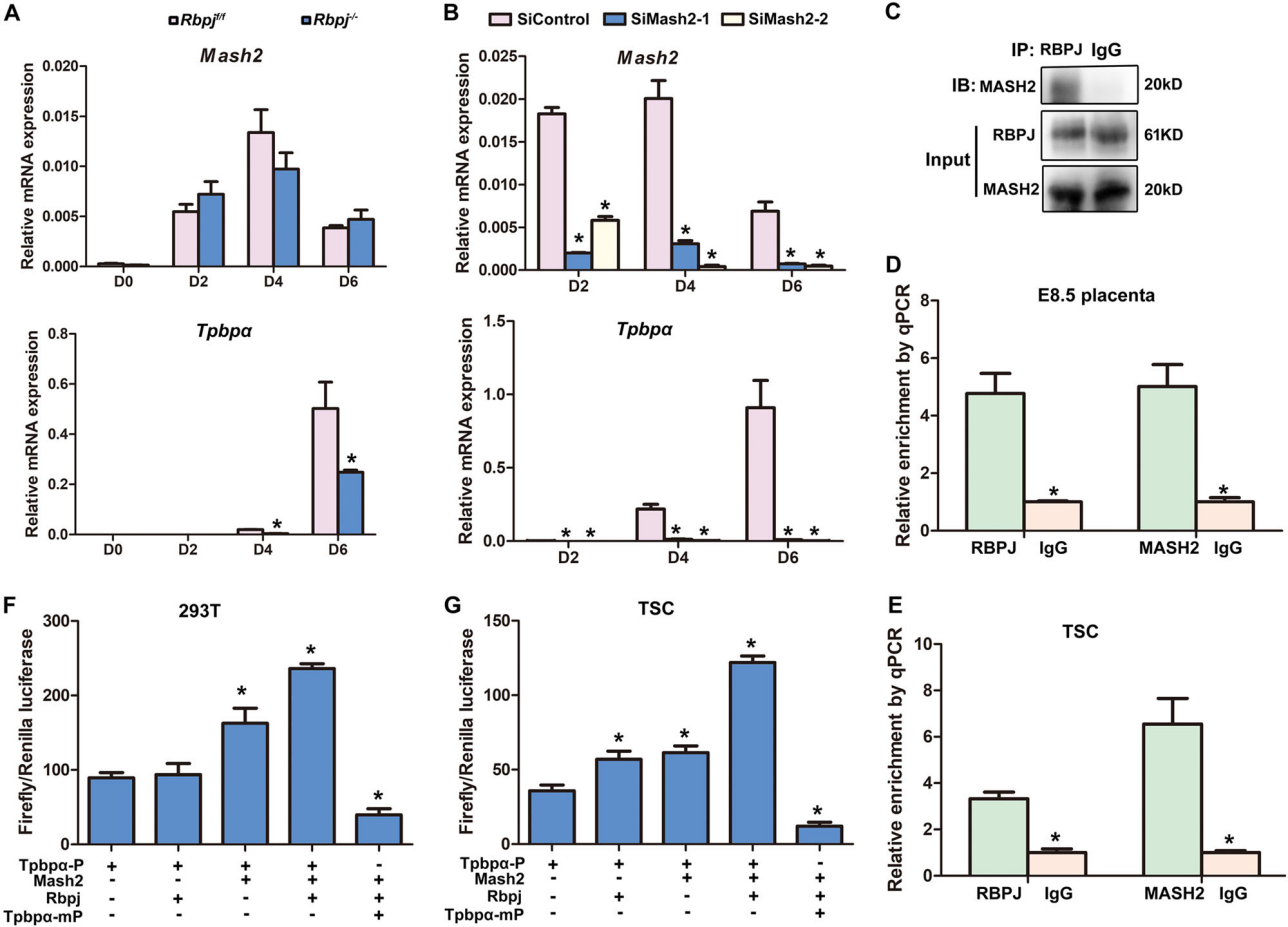
Rbpj facilitates Mash2 to promote the specification of *Tpbpα*-positive trophoblast. **a** Quantitative RT-PCR analysis of *Mash2* and *Tpbpα* during the differentiation of *Rbpj*^f/f^ and *Rbpj*^−/−^ trophoblast stem cells. **b** The expression of *Mash2* and *Tpbpα* after Mash2 knockdown via siRNA was detected by quantitative RT-PCR, during trophoblast stem cell differentiation. Values are normalized by GAPDH expression level and indicated as mean ± SEM. *N* = 3. *P < 0.05. **c** The interactions between the Rbpj and Mash2 were revealed by IP assay. **d**, **e** Rbpj and Mash2 were enriched at the promoter region of *Tpbpα* in both the E8.5 placentas (**d**) and in vitro-cultured trophoblast cells (**e**). **f**, **g** Rbpj facilitates Mash2 to activate the promoter activity of *Tpbpα* in both 293 T cells (**f**) and trophoblast stem cells (**g**), while deletion of Mash2 binding site in *Tpbpα* regulatory region diminished the activity of *Tpbpα* promoter increased by Rbpj and Mash2. *P < 0.05. Data in **a**–**g** are representative of at least three independent experiments

Previous evidence disclosed that Notch signaling regulated Mash2 through hairy/Enhancer of split (*Hes*) genes and Groucho genes (*Tle*) in *Drosophila*^33,34^, implying a potential coupling of Notch signaling with Mash2 during the specification of *Tpbpα*-positive cells. To reveal the underlying molecular mechanism governing normal specification of *Tpbpα*-positive trophoblast driven by Rbpj signaling, we further employed cultured trophoblast stem (TS) cell lines (Fig. S6a). Rbpj deletion did not perturb the stemness of trophoblast cells, since *Rbpj*^−/−^ trophoblast stem cells have been established successfully. We found that while the expression of *Mash2* was not disturbed, *Tpbpα* expression was decreased significantly, but not disappeared in Rbpj-deficient TS cells undergoing default differentiation in culture (Fig. 7a), consistent with in vivo observations (Fig. 6a). In addition, Mash2 knockdown by siRNA almost diminished *Tpbpα* expression (Fig. 7b), similar to the findings in Mash2 knockout mice^30^. These findings suggest that Rbpj might facilitate the functions of Mash2 during specification of *Tpbpα*-positive trophoblast. Co-immunoprecipitation analysis further revealed physical interaction between Mash2 and Rbpj in trophoblast cells (Fig. 7c). Since Rbpj and Mash2 are both transcription factors, we performed ChIP assay using antibodies against Mash2 and Rbpj, respectively. We found an enriched accumulation of both Mash2 and Rbpj in a trophoblast-specific regulatory region of *Tpbpα*, a 340-bp region that is 3.7 kb upstream of the TSS of *Tpbpα*^35^, in both E8.5 placenta (Fig. 7d) and TSC (Fig. 7e). These data demonstrate a functional association of Rbpj and Mash2 during the specification of *Tpbpα*-positive trophoblast cells.

Regarding the physiological significance of Mash2-Rbpj interaction, we employed luciferase assay to assess the activity of *Tpbpα* promotor in both HEK 293 T cells and TS cells. While Mash2 increased luciferase activity significantly, Rbpj alone exhibited no influence on luciferase activity in HEK 293 T cells (Fig. 7f), which have extremely low expression of endogenous *Mash2*. Importantly, co-transfection of Mash2 and Rbpj increased the activity of *Tpbpα* promoter significantly (Fig. 7f). In consistence, a similar phenomenon was observed in cultured TS cells undergoing differentiation; besides that, Rbpj alone was able to promote the activity of *Tpbpα* promoter (Fig. 7g), since TS cells expressed endogenous Mash2. Moreover, employing in silico analysis of the trophoblast-specific regulatory region of *Tpbpα*, we found the binding site of Mash2, but not that of Rbpj. When the binding site of Mash2 was deleted, the activity of *Tpbpα* promoter increased by Rbpj and Mash2 was decreased significantly (Fig. 7f, g). In addition, while endogenous *Tpbpα* expression was promoted by Rbpj alone in the presence of endogenous Mash2, its expression was further increased synergistically by Mash2 and Rbpj overexpression in differentiated trophoblast cells (Fig. S6b, c). These data demonstrate that Rbpj facilitates Mash2 to promote the specification of *Tpbpα*-positive trophoblast.

## Discussion

**Fig. 8:**
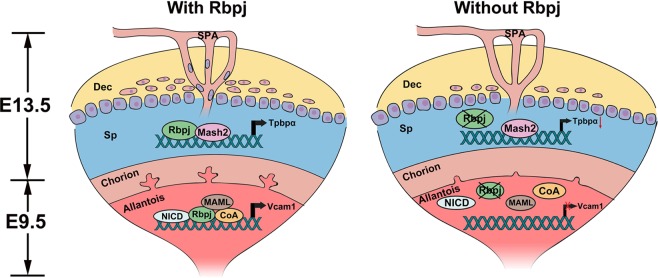
Diagram illustrating the functions of RBPJ-mediated signaling during chorioallantoic development and trophoblast differentiation in EPC (Sp). In the presence of Rbpj (with Rbpj), Rbpj regulates the expression of *Vcam1* in the allantois through a Notch-dependent manner to ensure normal chorioallantoic branching morphogenesis, and facilitated Mash2 in EPC (Sp) to promote the specification of *Tpbpα*-positive trophoblast cells simultaneously. However, failed chorioallantoic branching and defective trophoblast differentiation in EPC (Sp) were observed in the absence of Rbpj (without Rbpj). Dec decidual basalis, Sp spongiotrophoblast layer, SPA spiral arteries

Placenta acts as the interface between the mother and fetus for the exchanges of nutrients, gases, and wastes. During placental development, fetal vessels grow into the labyrinth via chorioallantoic branching morphogenesis. Simultaneously, the maternal blood enters the placenta through SPA remodeled by TGCs and facilitated by invasive glycogen trophoblast cells, and forms blood sinus in the placental labyrinth layer. We provided herein genetic and molecular evidence that allantoic Rbpj guides normal chorioallantoic fusion and branching morphogenesis by targeting *Vcam1*, while trophoblast Rbpj facilitates the functions of Mash2 during specification of *Tpbpα*-positive trophoblast, which are important for the differentiation and invasion of SPA-TGCs and glycogen trophoblast cells during placental development (Fig. 8). These findings highlight the necessity of a spatiotemporal coordination of Rbpj signaling for normal placental morphogenesis.

Chorioallantoic fusion is the first step for fetal vessels to invaginate into the chorion and followed by further branching of fetal vessels in the placental labyrinth. In this study, Rbpj-mediated signaling regulates chorioallantoic fusion via *Vcam1* in a Notch-dependent manner, highlighting the necessity of Rbpj-Vcam1 signaling during chorioallantoic fusion^23,36^. Moreover, we further demonstrated that this signaling pathway might also function during chorioallantoic branching beyond the initial fusion, since branching failed to develop further into the labyrinth in the absence of *Rbpj* or *Vcam1*. We found that only Vcam1 expression in allantois and allantois-derived fetal vessels was disturbed, but not that in fetuses with Rbpj deletion (Fig. 4b), suggesting that regulation of Vcam1 expression is tissue specific. Moreover, *Rbpj*-deficient mice with defective chorioallantoic fusion also suffered from impaired labyrinth development, with fetal vessels failing to extend and invaginate further into the labyrinth layer. We also found that cultured allantois exhibited decreased vascular remodeling, when Rbpj was deleted or Notch signaling was inhibited by DAPT (Fig. S7). These findings reinforce the content that allantoic Notch-Rbpj-Vcam1 signaling is also essential for remodeling of placental fetal vessels beyond the initial fusion^7,24,37^. In addition, Dll4 was reported to be expressed in fetal vascular endothelial cells of allantois and subsequent umbilical arteries. Haploid insufficiency of Dll4 exhibits similar phenotypes to that of *Rbpj*-null mice^38–40^, suggesting that Dll4 is a potential upstream driver of Rpbj-Vcam1 signaling governing normal vascular remodeling and the maintenance of fetal vessels during chorioallantoic morphogenesis.

During pregnancy, maternal blood flow through the placenta increases dramatically, largely due to significant vasodilation of blood vessels^10^. In mice, TGCs invading utero-placental SPA and glycogen trophoblast cells invading interstitially into the decidua, are associated with the remodeling of maternal SPA into dilated, inelastic tubes to increase maternal blood flow to the placenta^32^. It has been shown that TGCs and glycogen trophoblast cells are differentiated from progenitor cells localized within the ectoplacental cone, as well as the spongiotrophoblast layer later, and expressing spongiotrophoblast-specific gene *Tpbpα*^31^. In our work, we found that decreased *Tpbpα* expression by Rbpj deletion led to defective invasion of SPA-TGCs and glycogen trophoblast cells. This may help explain why fetal weight was not perturbed at E10.5, but was decreased at E13.5, in the presence of trophoblast-specific Rbpj deletion. Fetal weight at E10.5 was not disturbed, maybe due to normal development of placental labyrinth after *Rbpj* deletion. However, with rapid fetal growth since E10.5, the fetuses failed to get enough nutrients to meet their increasing needs, might attribute to impaired remodeling of maternal SPA and insufficient placental perfusion upon Rbpj deletion, leading to decreased fetal weight at E13.5.

Although it has been reported that Mash2 is a key determinant for specification of *Tpbpα*-positive trophoblast cells^41^, the mechanism controlling specialization of *Tpbpα*-positive cells remains largely unknown. In our study, we demonstrated that Rbpj facilitates Mash2 to promote *Tpbpα* expression and specialization of *Tpbpα*-positive cells. These findings provide a new line of evidence regarding the regulation of *Tpbpα*-positive trophoblast cells specification. Moreover, the findings that decreased *Tpbpα* expression upon Rbpj deletion led to defective invasion of SPA-TGCs and glycogen trophoblast cells are similar to the phenotypes of mice with conditional deletion of Notch2 via *Tpbpα*-Cre^42^. Although Notch2 is involved in the process during which *Tpbpα*-positive cells differentiate into invasive SPA-TGCs and glycogen trophoblast cells^42^, it seems that Notch2 does not participate in Rbpj-mediated signaling responsible for specification of *Tpbpα*-positive trophoblast cells in EPC, since the population of *Tpbpα*-positive cells was not disturbed in *Notch2*-deficient placentas^43^. Moreover, it has been shown that Rbpj could also function in a Notch-independent manner^18,44–46^, and trophoblast-specific inhibition of canonical Notch signaling did not disturb placental development^28^. We also found that Rbpj had no binding sites in the trophoblast-specific regulatory region of *Tpbpα*. Therefore, Rbpj-mediated signaling might regulate specification of *Tpbpα*-positive trophoblast cells in a noncanonical manner. In addition, it has been reported that trophoblast cells expressing Prdm/Blimp1 gave rise to SPA/endovascular TGCs, canal TGCs, and glycogen trophoblast cells, and *Tpbpα* expression was unaffected in the absence of Blimp1^47^, suggesting that Blimp1 might function after the specification of *Tpbpα*-positive trophoblast cells, and Rbpj signaling may also regulate specification of Blimp1-positive cells.

In summary, our study further shed light on the molecular network governing placental development and functions, and the findings might have clinical relevance, since abnormal Notch signaling contributes to pathogenesis of gestational diseases with aberrant placentation and trophoblast function in women^14,42^.

## Materials and methods

### Animals

*Rbpj*^*loxp*/*loxp*^ (*Rbpj*^f/f^) mice, *DNMAML*^*f*/*f*^ mice, and *Cyp19*^*cre*/+^ transgenic mice were generated as previously described^27,48,49^. Enhanced green fluorescent protein (*Egfp*)^*Tg*/+^, *Rosa26-LacZ*^f/f^, *Prm*^*cre*/+^, *ZP3-cre*^*cre*/+^, and *Sox2*^*cre*/+^ transgenic mice were obtained from Jackson Laboratory. *Rbpj*^f/f^ mice were mated with *Zp3*^*Cre*/+^ and *Prm*^*cre*/+^ to achieve systemic *Rbpj* deletion. *Rbpj*^f/f^ mice were mated with *Sox2*^*cre*/+^ and *Cyp19*^*cre*/+^ transgenic mice to get conditional deletion of *Rbpj* in the epiblast and trophoblast derivates, respectively. *DNMAML*^*f*/*f*^ mice were mated with *Prm*^*Cre*/+^ transgenic mice and *Cyp19*^*Cre*/+^ transgenic mice to silence canonical Notch signaling in the global and trophoblast-derived cells, respectively. Females were mated with fertile males of the same strain to induce pregnancy, and mice with virginal plus were considered as embryonic day 0.5 (E0.5). Mice were housed in the animal care facility of the Xiamen University according to the guidelines for the care and use of laboratory animals.

### Histological analysis and immunostaining

For hematoxylin and eosin staining, isolated implantation sites or whole dissected placental tissues were collected at various stages of gestation and fixed in 10% neutral buffer formalin. Then tissues were dehydrated and embedded in paraffin wax before being cut into 5-μm sections. For immunohistochemistry analysis, the rehydrated sections were incubated with RBPJ (1:200, Cell Signaling Technology) and Laminin (1:200, Sigma) antibodies, respectively. A Histostain-plus Kit (Zhongshan Golden Bridge Biotechnology) was used to visualize the antigen. For immunofluorescence, antibodies specific to Laminin (1:500, Sigma) and Vcam1 (1:500, Abcam) and secondary antibodies conjugated with Cy3 dyes (Jackson ImmunoResearch Laboratories) were used to stain rehydrated sections. Immunofluorescence images were captured in a Zeiss LSM 780 confocal scanning laser microscope.

### In situ hybridization

In situ hybridization was performed on cryosections as previously described^21,50^. Briefly, frozen sections (10 μm) were mounted onto poly-L-lysine-coated slides and fixed in 4% paraformaldehyde solution in PBS at 4 °C. After prehybridization, sections were hybridized at 45 °C for 4 h in 50% formamide buffer containing ^35^S or digoxygenin (DIG)-labeled sense or antisense RNA probes. The primers for probe production are listed in Table S1. After hybridization, sections were incubated with RNase A (10 mg/ml) at 37 °C for 20 min, and RNase A-resistant hybrids were detected by autoradiography, using liquid emulsion or NBT/BCIP chromogenic agent. Sections hybridized with the sense probes served as negative controls.

### Tetraploid aggregation assay

Tetraploid aggregation chimeras were generated as described previously with some modifications^51^. In brief, wild-type tetraploid embryos were generated by electrofusion of two-cell embryos derived from EGFP intercrosses. Fused embryos were cultured overnight in KSOM medium and developed to form four-cell embryos before the removal of zona pellucida with acidic Tyrode solution. Diploid *Rbpj*-null embryos at eight-cell or morula stage were collected at E2.5. Each diploid embryo with zona pellucida removed was aggregated with two tetraploid embryos. Aggregated chimeric embryos were allowed to develop into the blastocyst stage and then transferred into the pseudopregnant uteri of wild-type females. Chimeric embryos were dissected at E10.5. Both the placenta and embryo proper were visualized for EGFP under a dissecting microscope and subsequently fixed for staining with hematoxylin–eosin. The placental and embryo tissue was used as a DNA source for genotyping of diploid embryos.

### Quantitative real-time PCR

Total RNA was extracted from embryonic tissues or trophoblast cells using TRIzol reagent (Invitrogen), and 1–3 µg of total RNA after DNase treatment was used to synthesize cDNA. Quantitative RT-PCR analysis of different genes was performed using ABI QuantStudio 5 Real-Time PCR system, according to the manufacturer’s instructions. Assays were performed at least three times with each in duplicate. All the primers for real-time PCR are listed in Table S1.

### Plasmid construction, transfection, and luciferase assay

The promoter region of *Vcam1* gene (2 kb upstream relative to the transcription start site) with VP-BS1 and VP-BS2 wild-type versus mutant vectors (TGGGAA to TCTCAA) was generated using mouse genomic DNA as template and cloned into pGL3-basic vectors (Promega). Similarly, pGL3-basic vectors containing a trophoblast-specific regulatory region of *Tpbpα*, a 340-bp region that has been reported and is 3.7 kb upstream of the TSS of *Tpbpα*^35^, were constructed from genomic DNA similarly. Mutations of the Mash2 binding site were achieved by Fast Mutagenesis System (Stratagene). The coding sequence region for the intracellular domain of Notch1 and Notch2 were amplified from the mouse placenta and cloned into the pCDH expression vector. In addition, the coding sequence region of Rbpj and Mash2 was constructed with pEGFP-N1 and pcmv-myc expression vectors, respectively. Primers used are listed in Table S1.

HEK293T cells were maintained in Dulbecco’s Modified Eagle Medium (DMEM) (Hyclone) containing 10% fetal calf serum, 1 mM sodium pyruvate, 2 mM glutamine, 50 IU/ml penicillin, and 50 μg/ml streptomycin. All constructs were transiently transfected into HEK293T cells in 96-well plates, using Lipofectamine 2000 reagent (Invitrogen), according to the manufacturer’s instructions. pRLTK, the internal control plasmid expressing Renilla luciferase (Promega), was cotransfected into the cells to normalize firefly luciferase activity of the reporter plasmids. Transfected cells were harvested after 24 h. Luciferase assay was performed by Dual-Luciferase Reporter System (Promega) according to the manufacturer’s instructions. Assays were performed at least three times with each in duplicate.

### Allantois culture and immunostaining

Briefly, *Rbpj*^f/f^ and *Rbpj*^−/−^ pregnant females were killed by cervical dislocation at E8.0. Allantois could be clearly identified at the end of PS in the exocoelom after wiping off the Reichert’s membrane. The floating allantoises were excised at the basal part, rinsed in cold CZB and culture medium three times, respectively, transferred to the 24-well Petri dish that has been coated with fibronectin, and then cultured for 24 h. The allantois explants were fixed in 4% PFA, permeabilized in 0.2% Triton X-100, and incubated with rat anti-PECAM1 (1:500, BD) overnight at 4 °C. Then they were washed in 0.1% BSA–PBS, incubated with the Cy3-labeled secondary antibody.

### Co-immunoprecipitation (Co-IP) and chromatin immunoprecipitation (ChIP)

Protein samples (1 mg) from E8.5 placentas or trophoblast cells lysated by mild buffer (25 mM Tris, 150 mM NaCl, 1 mM EDTA, 1% Nonidet P-40, and 5% glycerol; pH 7.4) were used for Co-IP experiments. Antibodies to Rbpj (Santa Cruz Biotechnology), Mash2 (Merck) were used. Protein A agarose beads (Thermo-Fisher Scientific) were washed and incubated with protein lysates overnight at 4 °C. Immunoprecipitated proteins were separated by SDS/PAGE and detected by immunoblotting using the respective antibodies. ChIP analysis was performed according to the instructions of the ChIP Assay Kit (Millipore). In brief, E8.5 placentas or trophoblast cells were collected and suspended in 1% formaldehyde (Sigma)–PBS solution for cross-linking. After the cross-linking was terminated, cell pellets were lysed and sheared by sonication, until the average length of DNA was ∼500 bp as evaluated by agarose gel electrophoresis. The sheared chromatin fragments were incubated with the RBPJ (Santa Cruz Biotechnology) or Mash2 (Millipore) antibodies overnight at 4 °C. Normal goat IgG (Santa Cruz Biotechnology) was used as a negative control for nonspecific immunoprecipitation. After washing, the beads were suspended in elution buffer and the precipitated protein/DNA complexes were eluted from the antibodies/beads. Then the chromatin was subjected to cross-link reversal and DNA was purified by phenol/chloroform extraction and ethanol precipitation. Specific primers listed in Table S1 were used to detect immunoprecipitated chromatin fragments and input chromatin.

### Statistical analysis

Statistical analysis was performed with SPSS11.5 program. Comparison of means was performed using the independent-samples *t* test. Each experiment was performed at least three times with each in duplicate. Data were shown as means ± SEM. *p < 0.05; **0.01 < *p* < 0.05.

## Supplementary information

Supplementary Materials
