# Supplementary material for: Spatiotemporal coordination of trophoblast and allantoic Rbpj signaling directs normal placental morphogenesis
Source: Cell Death Dis. 2019 Jun 5;10(6):438. doi: 10.1038/s41419-019-1683-1 (PMC6549187; doi:10.1038/s41419-019-1683-1)
Supplement: Supplementary file 1 — Supplementary Materials [file 41419_2019_1683_MOESM1_ESM.pdf]

Supplementary Materials

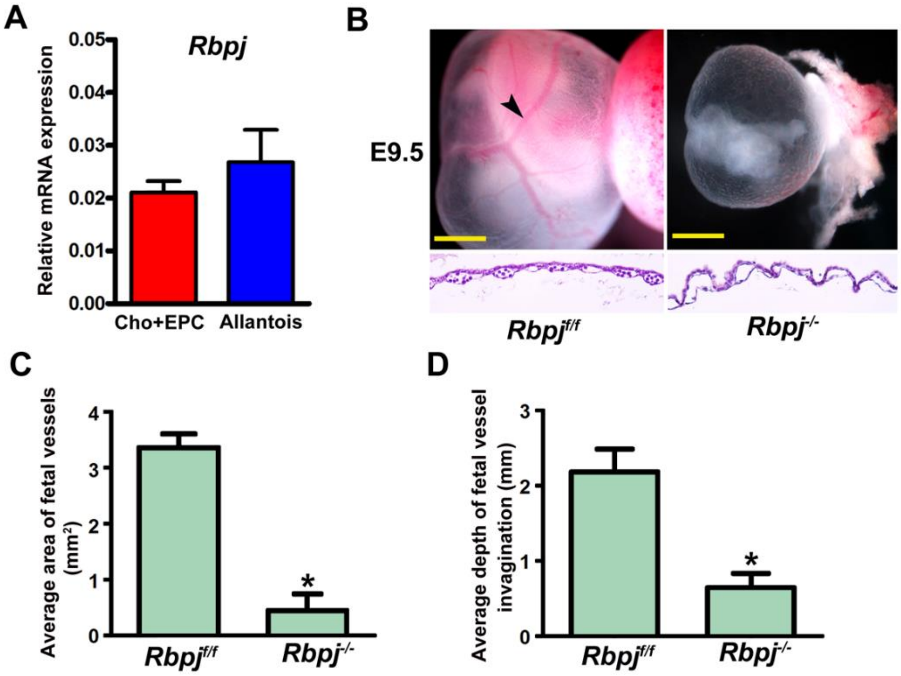

Figure S1 The expression of *Rbpj* and the phenotype of *Rbpj* deletion. (A) The expression of *Rbpj* mRNA in isolated allantois and chorion plus EPC was detected by quantitative RT-PCR. Values are normalized by GAPDH expression level and indicated as mean ± SEM. *N*=3. (B) Whole mount views of *Rbpj*$^{f/f}$ and *Rbpj*$^{-/-}$ placentas and yolk sacs at E9.5. Black arrowhead indicates large vitelline vessels in *Rbpj*$^{f/f}$ yolk sacs. (C) The average area of the fetal vessels in the labyrinth layer of *Rbpj*$^{f/f}$ and *Rbpj*$^{-/-}$ placentas. (D) The average depth of fetal vessel invagination into the labyrinth layer in *Rbpj*$^{f/f}$ and *Rbpj*$^{-/-}$ placentas. Data in (B-D) are representative of at least three independent experiments. *P<0.05. Yellow scale bars: 1mm.

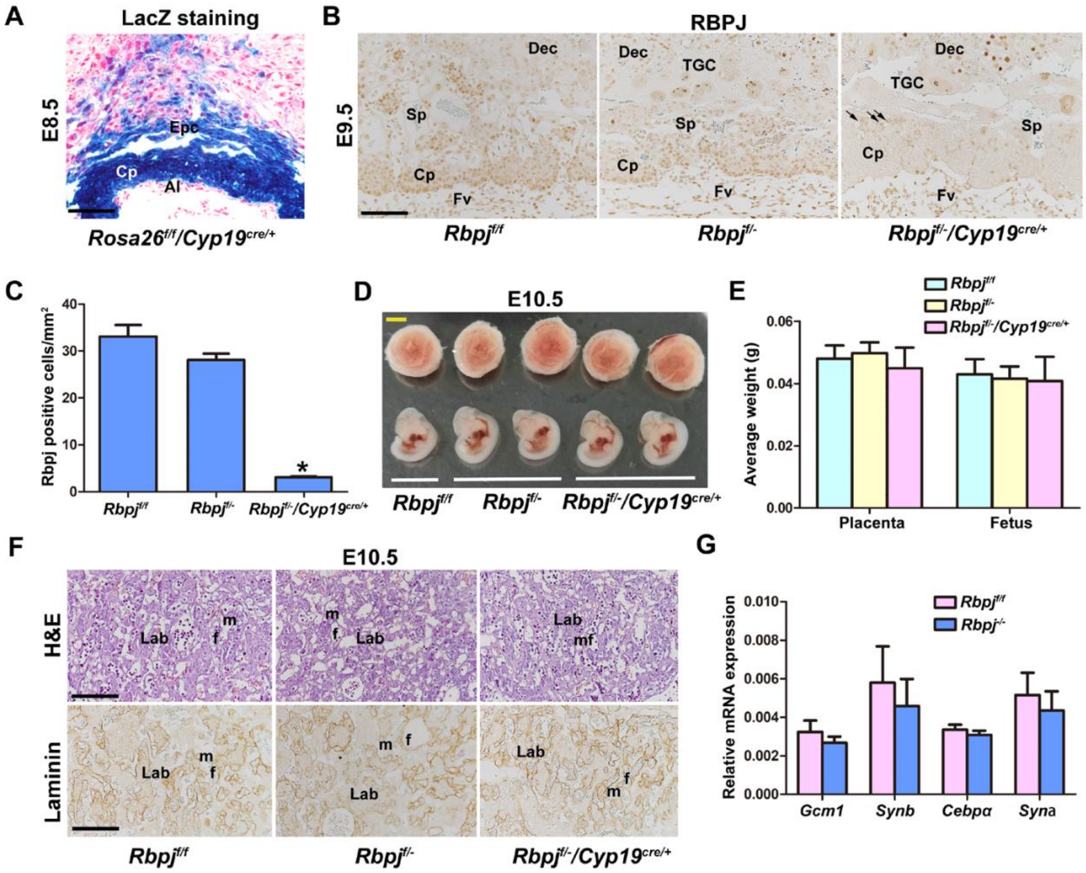

Figure S2 The efficiency of trophoblast-specific *Rbpj* deletion by *Cyp19*$^{cre/+}$ and the phenotype after Rbpj deletion. (A, B) The efficiency of trophoblast-specific deletion of *Rbpj* by *Cyp19*$^{cre/+}$ was detected by LacZ staining (A) in placentas from *Rosa26*$^{loxp/loxp}$ mice and *Cyp19*$^{cre/+}$ mice intercross or RBPJ antibody (B). Note that, compared to the control (*Rbpj*$^{f/f}$ and *Rbpj*$^{f/-}$), RBPJ was deleted efficiently in trophoblast cells by *Cyp19*$^{cre/+}$, while kept expression in fetal cells and maternal decidua (*Rbpj*$^{f/-}$/*Cyp19*$^{cre/+}$) (B). Arrows indicate fetal blood cells. (C) The quantification of Rbpj-positive trophoblast cells and represented as the number of Rbpj-positive trophoblast cells per millimeter. *N*=3. *P<0.05. (D, E) The image (D) and weight (E) of placentas and fetuses at E10.5. *N*≥7. (F) HE and Laminin staining of E10.5 placental labyrinth. (G) The expression of trophoblast marker genes in the E9.0 chorion was revealed by quantitative RT-PCR. Values are normalized by GAPDH expression level and indicated as mean ± SEM. *N*=4. Images are representative of at least three independent experiments. Al, allantois; Cp, chorion plate; Epc, ectoplacental cone; fv or f, fetal vessels; Sp, spongiotrophoblast layer; TGC, trophoblast giant cells. Black scale bars: 100 µm; Yellow scale bars: 1mm.

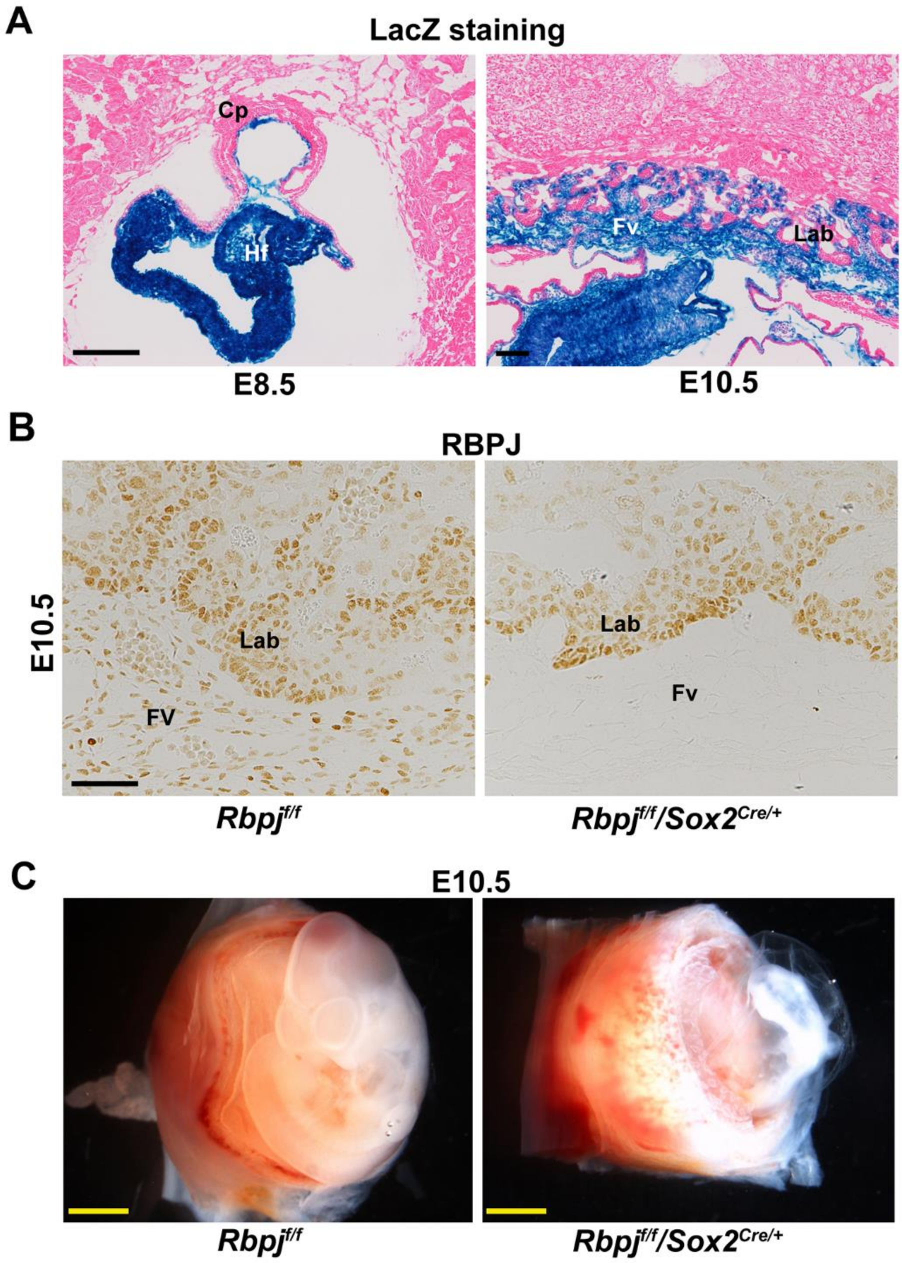

Figure S3 The embryonic-specific deletion of *Rbpj* by *Sox2$^{cre/+}$*. (A, B) The efficiency of conditional deletion of *Rbpj* in allantois (derived from epiblast) was detected by *Sox2$^{cre/+}$* via LacZ staining (A) in placentas from *Rosa26$^{loxp/loxp}$* mouse and *Sox2$^{cre/+}$* mouse intercross or RBPJ antibody (B). Note that RBPJ was deleted efficiently in fetal vessels (derived from allantois), while kept expression in trophoblast cells (B). (C) Growth arrest was revealed in conceptus with allantois-specific deletion of *Rbpj* by *Sox2$^{cre/+}$*. Images are representative of at least three independent experiments. Cp, chorionic plate; Hf, head fold; Lab, labyrinth; Fv, fetal blood vessel. Yellow scale bars: 1 mm; black scale bars: 100 µm.

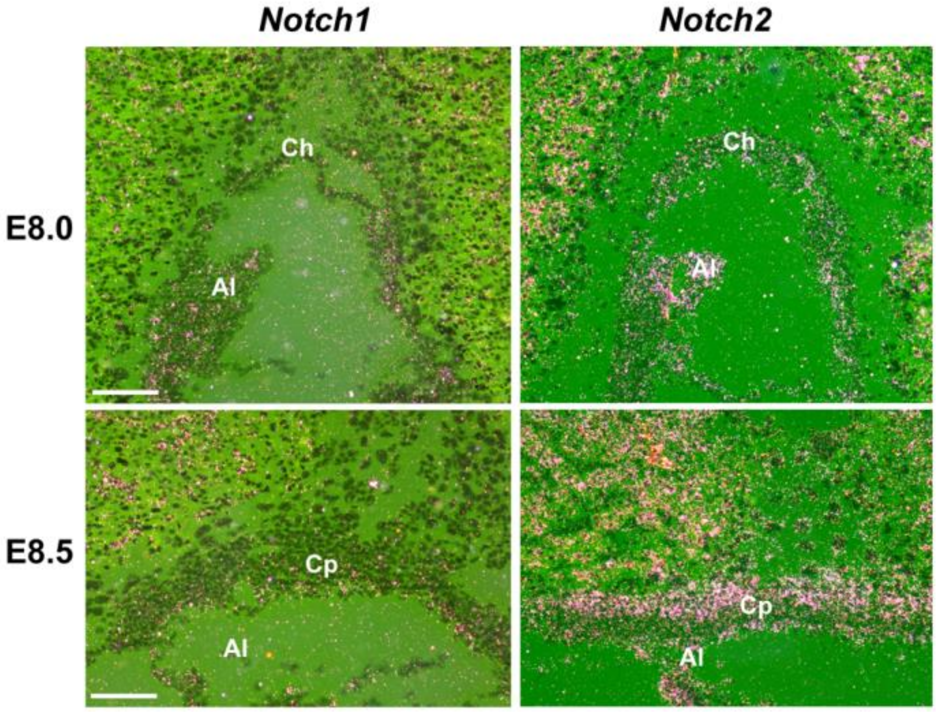

Figure S4 The localization of Notch1 and Notch2 during chorioallantoic fusion. In situ hybridization analysis revealed that Notch1 and Notch2 are both expressed in the chorion and allantois at E8.0 and E8.5, before and after the fusion of chorion and allantois. Images are representative of two independent experiments. Al, allantois; Ch, chorion plate; Cp, chorion plate. Scale bars: 100μm.

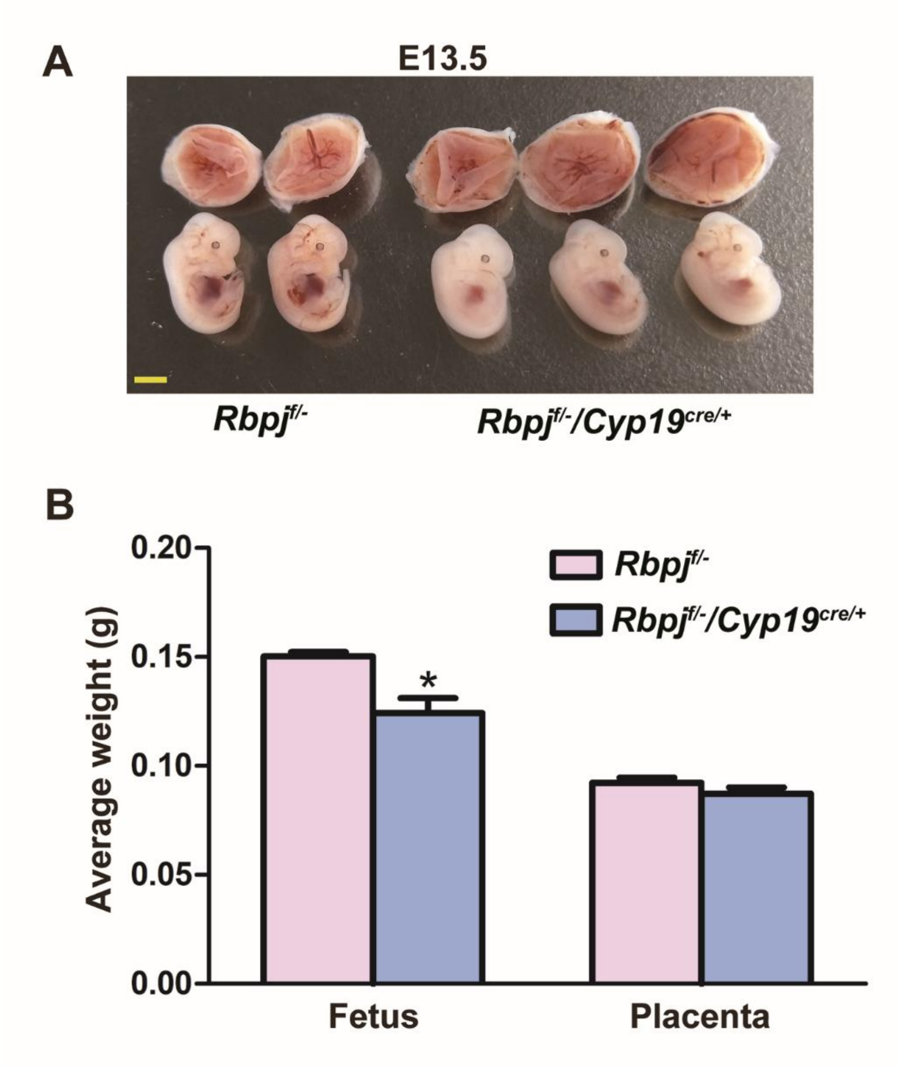

Figure S5 Trophoblast-specific deletion of *Rbpj* reduced fetal weight at E13.5. (A, B) The images (A) and weight (B) of placentas and fetuses with trophoblast-specific deletion of Rbpj by *Cyp19*$^{Cre/+}$. Data in (A) are representative of at least three independent experiments, *N*=8 in (B). *P<0.05. Yellow scale bars: 1mm.

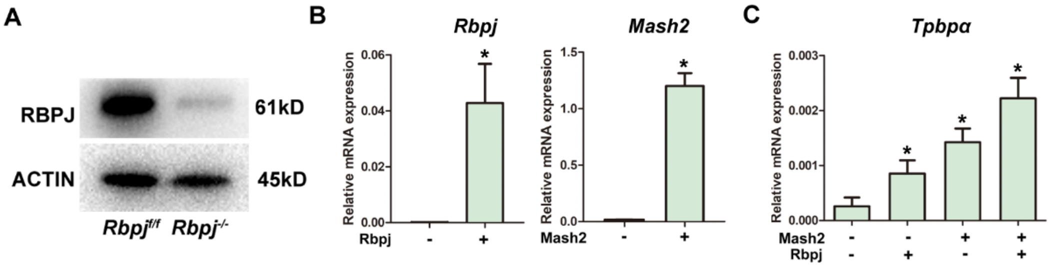

Figure S6 Rbpj facilitates Mash2 to promote *Tpbpα* expression in differentiated trophoblast cells. (A) The expression of Rbpj was detected by Western blot in *Rbpj*$^{f/f}$ and *Rbpj*$^{-/-}$ trophoblast stem cells. (B) The mRNA expression of Rbpj and Mash2 was analysed by quantitative RT-PCR after overexpression of Rbpj or Mash2 in cultured trophoblast cells differentiated for 48h (Day 2). (C) The expression of *Tpbpα* mRNA in the presence of Rbpj and Mash2, or the both was revealed by quantitative RT-PCR. Values are normalized by GAPDH expression level and indicated as mean±SEM. Data are representative of three independent experiments. *P<0.05.

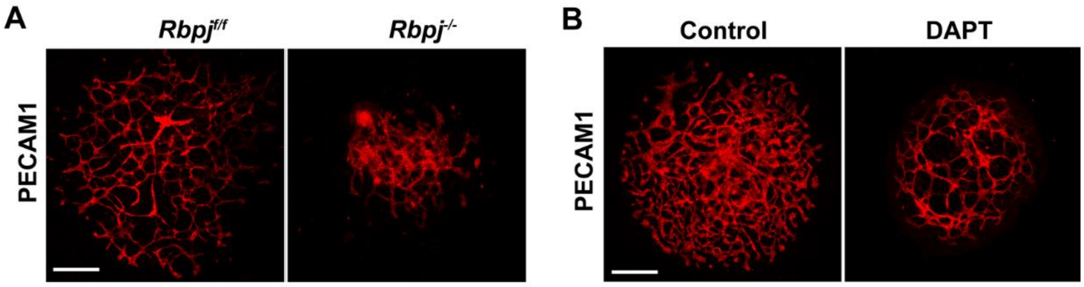

Figure S7 Rbpj deletion or inhibition of canonical Notch signaling decreased allantoic vascular remodeling in vitro. (A) Rbpj deletion decreased allantoic vascular remodeling. (B) Inhibition of Notch signaling with DAPT disturbed allantoic vascular remodeling. Cy3-labeled PECAM1 was in red. Images are representative of three independent experiments. Scale bars: 100µm.

Table S1 Primer information.

Name	Sequence (5'→3')
**Quantitative qRT-PCR primers**	
Rbpj-F	AGAGGAGTTCACAGTTAGA
Rbpj-R	GTAATGCCGTCTGCTTAT
Ascl2 (Mash2)-F	CAGCTGCGAGGGAGAGCTAA
Ascl2 (Mash2)-R	GATGCTCAGTAGCCCCCTAACC
Tpbpα-F	CAGAGAGTGGCGATGGGTTTT
Tpbpα-R	GACAATGGCACAGTGGCTGTT
Gcm1-F	CCCCAGCAAGTTCCATCAGA
Gcm1-R	AAGGCTCACCTCCCGGATT
Synb-F	CCACCACCCATACGTTCAAA
Synb-R	GGTTATAGCAGGTGCCGAAG
Cebpα-F	AAAGCCAAGAAGTCGGTGGAC
Cebpα-R	CTTTATCTCGGCTCTTGCGC
Syna-F	AGATACCCCGATGACCACGTC
Syna-R	TGAGGATCGTCTGGGTGGAG
**Primers for in situ hybridization**	
Rbpj-F	CACCAGCCTTACCTTCACCT
Rbpj-R	TGCATTCAAGTCAGTTTCAGT
Vcam1-F	ATAGACAGCCCACTAAAC
Vcam1-R	TCAATGACGGGAGTAAA
Itga4-F	TGGCTCTATCGTGACTTGTGG
Itga4-R	CGACTTCGGTAGTATGTGGAC
Pl1-F	TTCCTCACTTGGAGCCTACA
Pl1-R	CTCTACATAACTGAGGAGGG
Tpbpa-F	TCCAAGGACCTCTGAAGAGC
Tpbpa-R	AGGATCCCACTTGTCAGGGG
Plf-F	TGAGGAATGGTCGTTGCTTT
Plf-R	TTTATGTCTGTGGCTTTGGA
Synb-F	CTTTCCACCACCCATACGTT
Synb-R	TGACCTTGAAGTGGGTAGGG

Cebpa-F	CGCTGGTGATCAAACAAGAG
Cebpa-R	GTCACTGG TCAACTCCAGCA
Syna-F	TTGCAATCACACCTTTCAGC
Syna-R	TGGTGTCCACAGACAGGGTA
Notch1-F	CCTTGGGTTCATGGATTAGT
Notch1-R	TCTGTCCTCAGTTGGATTTG
Notch2-F	GAGACATACGTGTGAGGAGA
Notch2-R	TTTTGCCAACCGGGACATCA
**Primers for the analysis of promoter activity**	
Vcam1-P-F	GGTACCGTGACTCATGATAATGGGTC
Vcam1-P-R	AAGCTTCCTTCTTTGTGCCCCACGTG
Vcam1-P-Mutant-F	AGCTGAAGGGGTTAACAGACGGACTTGG
Vcam1-P- Mutant-R	CTGTTAACCCCTTCAGCTGCTTTCTGGG
Tpbpα-P-F	GTAGACTGTTCCTCAGTAGATTG
Tpbpα-P-R	CTCGAGAGAGAAAGACACTTG
Tpbpα-P-Mutant-F	TAGGGAGGACTAGGTAAGGACAGAAGTTTATGCTAAACTG
Tpbpα-P-Mutant-R	TCCTTACCTAGTCCTCCCTACGTGATCAAGAG
**Primers for the ChIP assay**	
Tpbpα-F-1	GTAGACTGTTCCTCAGTAGATTGAAAATTG
Tpbpα-R-1	CTATTATGTCAGTTTAGCATAAACTTCTGTCC
Tpbpα-F-2	GCTAAACTGACATAATAGAATTGTTGGCTG
Tpbpα-R-2	CTCGAGAGAGAAAGACACTTGTTGTAAG
